# Non-coding RNAs regulate mitochondrial dynamics in the development of gastric cancer

**DOI:** 10.3389/fmolb.2023.1107651

**Published:** 2023-01-12

**Authors:** Xiatian Chen, Chuang Wei, Liting Huang, Konstantinos Syrigos, Yuzhen Li, Peifeng Li

**Affiliations:** ^1^ Institute of Translational Medicine, The Affiliated Hospital of Qingdao University, Qingdao University, Qingdao, China; ^2^ School of Basic Medicine, Qingdao University, Qingdao, China; ^3^ National and Kapodistrian University of Athens, Athens, Greece; ^4^ Basic Medical Department, Graduate School, Chinese PLA General Hospital, Beijing, China

**Keywords:** non-coding RNA, mitochondrial dynamic, gastric cancer, therapeutic strategy, cancer progression

## Abstract

Gastric cancer (GC) is a malignant cancer that reduces life expectancy worldwide. Although treatment strategies have improved, patients with GC still have poor prognoses. Hence, it is necessary to understand the molecular mechanisms of GC and to find new therapeutic targets. Mitochondrial dynamics and mitochondrial dysfunction are associated with cancer cell growth and progression. Numerous studies have reported that non-coding RNAs (ncRNAs) can participate in the occurrence and development of GC by regulating mitochondrial dynamics. Elucidating the crosstalk between ncRNAs and mitochondria would be helpful in preventing and treating GC. Herein, we review and summarize the functions of oncogenes and tumor suppressors in suppressing ncRNAs and regulating mitochondrial dynamics in GC tumor growth, proliferation, invasion and metastasis. This review provides new insights into the pathogenesis of and intervention for GC.

## 1 Introduction

Gastric cancer (GC) is a malignant cancer with the fifth highest incidence and fourth highest mortality in the world (Sung et al., 2021). The pathogenesis of GC is related to several factors, including dietary, infectious, genetic, and epigenetic alterations. Chronic *Helicobacter pylori* infection is the leading cause of non-cardia GC (Plummer et al., 2015), but most patients with early GC have no obvious symptoms; diagnosed patients are usually in the intermediate and advanced stages of GC, and have a 5-year survival rate below 30% (Wei et al., 2022). The proliferation and metastasis of tumor cells require large amounts of material and energy to support tumorigenesis. Mitochondria serve as metabolic centers, generating energy through oxidative phosphorylation in eukaryotic cells (Nunnari and Suomalainen, 2012). Mitochondrial dynamics refer to how mitochondria change their shape, distribution, size, and function through the dynamic balance between continuous fission and fusion to meet the energy and product requirements of host cells and to respond to environmental changes. Mitochondrial fusion allows gene products to be transferred between mitochondria for optimal functioning. In contrast, fission is crucial for mitochondrial division and quality control. In addition, mitochondrial dynamics are linked to cellular activity, such as cell cycle, apoptosis, and Ca^2+^ signaling (Giacomello et al., 2020; Peng et al., 2020; Schell et al., 2020), and studies have demonstrated that mitochondrial dysfunction is related to the occurrence and development of GC (Ng et al., 2021; Poole and Macleod, 2021). Therefore, reviewing and understanding the dynamic regulation of and functional changes in mitochondria is important for the clinical treatment of GC.

RNAs are regarded as transmitters of genetic information and with the development of molecular technology, the understanding of RNAs has improved (Dutta et al., 2021) About 80% of the human genome is transcribed into RNAs, but non-coding RNAs (ncRNAs) account for the majority (Zhang et al., 2019; Zhou et al., 2022). According to length of RNAs, regulatory ncRNAs can be further divided into two categories: small ncRNAs with lengths less than 200 nucleotides, including microRNAs (miRNAs) and PIWI-interacting RNAs (piRNAs), and long non-coding RNAs (lncRNAs) with lengths over 200 nucleotides. In addition, another class of ncRNA with a covalently closed circular structure [circular RNAs (circRNAs)] have emerged as critical regulator of gene expression. Even the ncRNAs are not involved in coding proteins, play important roles in many cellular activities. Many studies have shown that ncRNAs are expressed abnormally in many diseases, such as heart disease and cancer (Zhou et al., 2018; Gowda et al., 2022; Ren et al., 2022). NcRNAs can regulate the expression of other endogenous competing RNAs, regulating transcription gene translation and protein localization.

Some research has shown that ncRNAs contribute to the synchronization of a series of important cellular and mitochondrial biological processes (Song et al., 2019; Zhang et al., 2021). Some ncRNAs involved in the signal pathways of GC have numerous crosslinks with those involved in mitochondria. The targeted regulation of ncRNAs in genes is closely related to mitochondrial function. NcRNAs play a vital biological role by directly influencing mitochondrial dynamics in cardiovascular diseases (Aung et al., 2021). Therefore, it is important to understand the crosstalk between ncRNAs and mitochondrial functions for GC prevention and treatment. In this study, we reviewed the role of ncRNAs in regulating mitochondrial dynamics in the pathological process of GC. This work may provide new insights for further investigation into the invasion and proliferation mechanisms of GC and provide some promising molecularly targeted therapies.

## 2 NcRNA biogenesis and functions

**FIGURE 1:**
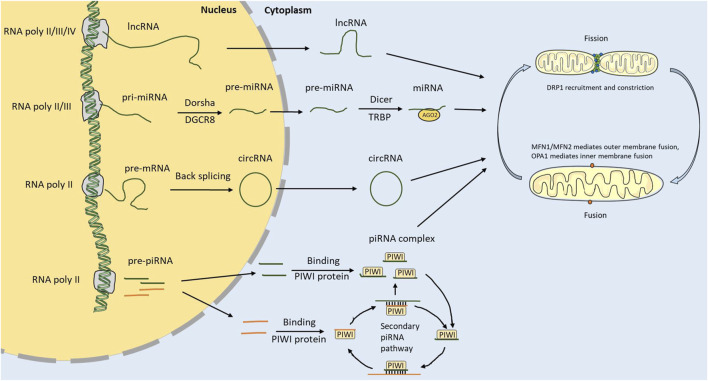
ncRNA biosynthesis and regulation of mitochondrial dynamics. ncRNA is transcribed by RNA polymerase in the nucleus. lncRNAs and circRNAs can be transported out of the nucleus through nuclear pores. pri-miRNAs are cleaved into pre-miRNAs by DGCR8 and Dorsha in the nucleus, then transported out of the nucleus and cleaved into miRNAs by Dicer and TRBP. The strand miRNAs attach to AGO2 to form the RISC. The piRNAs are transcribed from the piRNA cluster. Mature piRNAs can be synthesized through the primary and secondary piRNA pathways. ncRNA can regulate the mitochondrial dynamics by regulating core genes related to fusion and fission.

NcRNAs are synthesized in the nuclei and play a biological role in the cytoplasm. Because they cannot encode proteins, ncRNAs have long been regarded as “transcriptional waste products” of transcriptase II. Studies discovered that these “waste products” participate in nearly all life processes, such as cell growth and death (Slack and Chinnaiyan, 2019; Zhao X. et al., 2020; Sati and Parhar, 2021) (Figure 1).

miRNAs of 22 nucleotides in length are the earliest most widely studied ncRNAs (Shukla et al., 2011). Mature miRNAs covalently bind mRNAs to induce degradation or inhibit their expression by forming silencing complexes with specific protein compositions (Ha and Kim, 2014). miRNAs are synthesized through RNA polymerase II/III, transcribing genes containing miRNA sites to form primary miRNAs with two stem-loops (Mousavi et al., 2022). The DiGeorge syndrome critical region 8 (DGCR8) and Drosha subsequently cut the primary miRNAs, resulting in a single stem-loop structural precursor with approximately 70 nucleotides. The precursor miRNAs cross the nuclear envelopes into the cytoplasm through Ran-GTP-Exportin-5 transporters. Precursor miRNAs are recognized and processed by the transactivation response element RNA-binding protein (TRBP) and dicer in the cytoplasm. The modified precursor miRNAs separate into form two strands: one strand that rapidly degrades, and another that is bound by argonaute 2 (AGO2) protein to form RNA-induced silencing complex (RISC), which can bind to the 3′UTR of mRNAs and affect their expression.

piRNAs are 24–31 nucleotides long and mainly derive from transposons, coding regions and specific intergenic sites in the genome (Iwasaki et al., 2015). The function of piRNAs is to protect the genome from transposons, especially germ cells. Studies have shown that although their expression is low in somatic cells, they have additional functions therein (Ortogero et al., 2014; Ozata et al., 2019). They play a functional role in posttranscriptional gene silencing by forming functional complexes with PIWI proteins (Iwasaki et al., 2015). Generally, two piRNA-producing pathways are conserved in species, from sponges to higher mammals (Yamashiro and Siomi, 2018; Ozata et al., 2019; Chen S. et al., 2021). The primary processing pathway occurs in somatic cells and consists of single-stranded piRNA clusters, double-stranded piRNA clusters, gene-derived piRNAs, and transposon-derived generation pathways. Single-stranded piRNA precursors are processed into piRNA intermediates that bind to PIWI proteins and undergo nuclear or cytoplasmic silencing. The secondary processing pathway, also called the ping-pang pathway, is related to the posttranscriptional silencing functions of piRISC. In this pathway, the main piRISC proteins are AUB and AGO3. AUB binds to a piRNA to form a complex that recognizes and silences the mRNA. Secondary piRNAs are generated through transcript, cleavage, and then shorter cleaved product is loaded into the AGO3 protein, forming an AGO3-piRNA complex precursor. The mature AGO3-piRNA complex recognizes and cleaves the piRNA precursor transcript, and the cleaved product can initiate a new cycle to continuously produce substrates.

LncRNAs are remarkably similar to mRNAs (Kanduri, 2016; Quinn and Chang, 2016). Their transcriptional genomic sites are similar to mRNA transcriptional genomic sites in chromatin status, and they are usually capped, spliced, and polyadenylated at the 5’ ends. In most cases, except for untranslated open reading frames, there is no biochemical difference between lncRNAs and mRNAs, but there are also many differences. For instance, lncRNAs are shorter in length than mRNAs with fewer exons. Moreover, lncRNAs have relatively low expression levels and poor first-order sequence conservation. Several studies have revealed that lncRNAs can function as competing endogenous RNAs to bind with miRNAs and circRNAs (Guo C. J. et al., 2020; Statello et al., 2021). LncRNA can also bind to mRNAs and specific protein sites. In addition, lncRNAs can function as precursors for some small molecules that target RNA.

circRNAs were first discovered in plants and suspected to be a pathogenic viroid (Sanger et al., 1976). Later studies found that circular RNA molecules were also distributed in prokaryotes, eukaryotes, and mammals (Capel et al., 1993; Ford and Ares, 1994). With the help of high-throughput sequencing and bioanalysis techniques, cirRNAs have gradually been identified and found to play an important role in biological processes. circRNAs are not easily degraded by exonuclease due to their ring structure, which makes them more stable than linear RNAs (Zhu et al., 2022). A large number of circRNAs exist in the cytoplasm of eukaryotic cells, mainly derived from exons, and small numbers of intron-derived circRNAs exist in the nuclei. circRNAs are generated from the back-splicing of precursor mRNAs and are divided into three types according to the positions of their transcription loops: intronic RNAs, exonic circRNAs, and exon-intron circRNAs. Exonic circRNAs account for the majority (Tang et al., 2021). circRNA rings are formed in two main ways. In the first, lariat RNA is generated by the intron during splicing, which is formed by a 2′,5′-phosphodiester bond between the 5′splice site and the branch site of the intron, and a loop is formed by the exon of the gene when the downstream 5′splice site forms a 3′,5′phosphodiester bond with the upstream 3′splice site. The second type of ring formation mainly depends on the complementary pairing of repeated sequences of introns or the interactions between specific RNA-binding proteins. circRNAs have many functions, including binding to related binding proteins as transcriptional regulators, acting as miRNA sponges, and generating pseudogenes (Yang et al., 2021; Liu and Chen, 2022). Most circRNAs are non-coding, but a few contain internal ribosome entry site sequences (IRESs) that enable them to translate (Legnini et al., 2017; Biswas et al., 2021).

## 3 Altered mitochondrial dynamics associated with tumorigenesis

The earliest research found that cancer absorbs and ferments glucose to form lactic acid under aerobic conditions, so mitochondrial respiratory defects are the basis of cancer (Warburg, 1956). However, later studies found that not all cancers have this glycolytic feature; most still retain mitochondrial function (Vasan et al., 2020). Some cancer cells are robustly involved in the glycolysis and TCA cycle metabolism processes in mitochondria (DeBerardinis and Chandel, 2016). The increased rate of the metabolic process can activate various oncogenic controllers related to many signaling pathways. Tumor cells lost their mitochondrial ubiquinone regeneration ability, and the mitochondrial complex III subunit is a crucial part of tumor cell proliferation (Wang et al., 2020). Therefore, functional electron transport chain is required to oxidize ubiquinol for tumor growth (Meyers et al., 2017). Metabolites produced by the TCA cycle synthesize nucleotides, lipids, amino acids, and heme. In addition, the cycle produces oncogenic metabolites in some tumor contexts (Birsoy et al., 2015). Although some tumors rely only on glycolysis to meet their biological needs, they still need products from the mitochondrial oxidation process to promote cell proliferation (Sullivan et al., 2015). Synthetic requirements and the production of oncogenic metabolites promote tumorigenesis (Cluntun et al., 2017). Moreover, mitochondria are the signal transmission centers in cells, participating in cell apoptosis, autophagy, calcium transport, and other processes (Averbeck and Rodriguez-Lafrasse, 2021). Mitochondrial dynamics help maintain the mitochondrial pool and optimal mitochondrial oxidative phosphorylation activity in cells, allowing efficient transport and distribution of mitochondrial content.

### 3.1 Mitochondrial fission

The maintenance of normal mitochondrial function depends on a balance between mitochondrial fission and fusion, but this dynamism is dysregulated in most cancers. Mitochondrial fission is the division of a mitochondrion into two mitochondria. This process is important for maintaining mitochondrial numbers, distribution, and cell apoptosis activation (Tait and Green, 2010). Mitochondrial fission is related to the activation of dynamin-related protein 1 (DRP1) and mitochondrial fission one protein (FIS1) (Westermann, 2010; Wai and Langer, 2016). DRP1 is an evolutionarily conserved GTPase in approximately 97% of cytoplasm, and it lyses mitochondria by localizing the adaptor protein FIS1 in the mitochondrial membrane. Other proteins, such as dynamin 2, human mitochondrial dynamics protein 49 (MID49), and MID51 are also related to the fission process (Chan, 2012). Abnormal DRP1 expression has been identified in various human cancers, and its upregulation promotes the growth and metastasis of pancreatic cancer cells through increased mitochondrial fission and aerobic glycolysis (Liang et al., 2020). High glucose levels can increase the expression of DRP1, resulting in cell dysfunction and promoting the migration and invasion of endometrial cancer cells (Guo J. et al., 2020). The high expression of DRP1 was correlated with poor survival of head and neck cancer patients (Huang T. L. et al., 2022). DRP1 deletion can affect aerobic glycolysis and suppress tumor growth and metastasis (Huang T. L. et al., 2022). FIS1 is overexpressed in GC, and its expression is correlated with cancer metastasis (Karimi et al., 2022). Fan et al. (Fan et al., 2015) found that FIS1 knockdown could reduce mitochondrial fission and cisplatin sensitivity.

### 3.2 Mitochondrial fusion

Mitochondrial fusion is the process by which two mitochondria join to become one. Previous studies have found that different regulatory molecules are required for the fusion of the inner and outer membranes of mitochondria (Mattie et al., 2018; Adebayo et al., 2021). The major proteins regulating outer membrane fusion are mitofusin 1 (MFN1) and mitofusin 2 (MFN2) (Hales and Fuller, 1997). Optic atrophy 1 (OPA1) is located in and participates in inner mitochondrial membrane fusion. Mitochondrial fusion is necessary for exchanging genetic material and maintaining normal function. In addition, the fusion process can be triggered by some treatments, such as autophagy and starvation (Gomes et al., 2011; Rambold et al., 2011). Mitochondrial fusion is overactivated in liver cancer and cholangiocarcinoma. OPA1 inhibition can effectively reduce the proliferation and migration of breast cancer cells (Zamberlan et al., 2022). MFN2 has previously been reported to regulate cell proliferation, apoptosis and differentiation (Papanicolaou et al., 2012; Gong et al., 2015). Xu et al. (Xu et al., 2017) found that MFN2 knockout promoted cell viability, colony formation, and invasion of breast cancer cells. GC patients with high levels of MFN2 have a worse overall survival rate, and these high levels could be prognostic markers for GC (Fang et al., 2017).

## 4 ncRNAs control mitochondrial dynamics in GC

**FIGURE 2:**
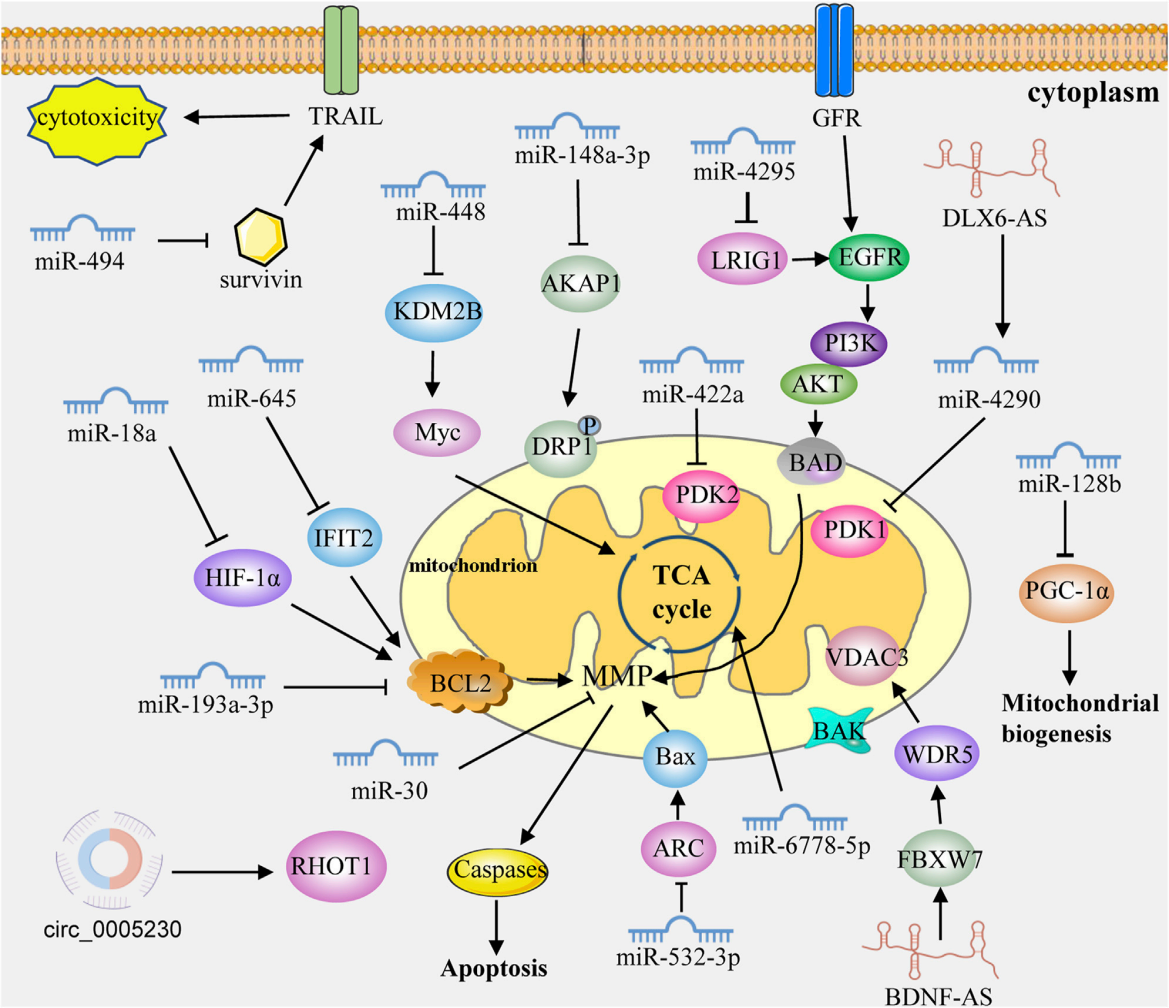
ncRNAs control mitochondrial dynamics in GC cells. ncRNAs can regulate mitochondrial functions and participate in GC through the direct or indirect targeting of the mitochondria-related genes or pathways. Caspases: caspase family protein (caspase3, 9 and 7). MMP: mitochondrial membrane permeabilization. GFR: growth factor receptor.

Mitochondria are important organelles in cells and hubs of many important biological processes. As intracellular gene regulators, ncRNAs can regulate mitochondrial functions and participate in GC through the direct or indirect targeting of the mitochondria-related genes or pathways (Figure 2; Table 1).

**TABLE 1:** Dysregulated ncRNAs regulate the mitochondrial function in GC.

Name	Type	Expression	Signaling	Mechanisms or functions of ncRNAs	References
miR-645	miRNA	up	IFIT2	Inhibits cell apoptosis by targeting IFIT2 and decreases the caspase 3/7 activity	Feng et al. (2014)
miR-18a	miRNA	down	HIF-1α	Inhibits cell apoptosis and invasion by targeting HIF-1α and suppressing the expression levels of mitochondrial apoptosis-related genes	Wu et al. (2015)
miR-448	miRNA	down	KDM2B/Myc	Promotes the proliferation and glycolysis metabolism through the KDM2B-Myc axis	Hong et al. (2016)
miR-148a-3p	miRNA	down	AKAP1/p53/DRP1	Serves as a biomarker; increases cisplatin resistance and inhibits fission by regulating AKAP1 expression and p53-mediated DRP1 dephosphorylation	Li et al. (2017)
miR-30	miRNA	up	p53	Inhibits p53-mediated mitochondrial apoptotic pathway and promotes proliferation	Wang et al. (2017)
miR-422a	miRNA	down	PDK2	Promotes proliferation and migration and suppresses the activity of PDH by targeting PDK2	He et al. (2018)
miR-494	miRNA	down	survivin	Suppresses TRAIL-induced mitochondrial collapse and the apoptosis pathway by targeting survivin	Xu et al. (2018)
miR-4295	miRNA	up	LRIG1/EGFR/PI3K/Akt	Promotes proliferation and inhibits cisplatin-induced apoptosis through the EGFR/PI3K/Akt signaling pathway by targeting LRIG1	Yan et al. (2018)
miR-193a-3p	miRNA	up	SRSF2	Inhibits mitochondrial apoptosis pathway and increases cisplatin resistance by targeting SRSF2	Lee et al. (2019)
miR-128b	miRNA	down	PGC-1α/SNAL1	Promotes mitochondrial biogenesis and cancer growth by regulating the PGC-1α-SNAL1 axis	Wang et al. (2019)
miR-6778-5p	miRNA	up	SHMT1/YWHAE	Regulates cytosolic one-carbon folate metabolism by targeting YWHAE	Zhao M. et al. (2020)
miR-532-3p	miRNA	down	ARC/Bax	Regulates mitochondrial damage and apoptosis by targeting ARC	Chen Z. et al. (2021)
DLX6-AS1	lncRNA	up	miR-4290/PDK1	Regulates tumor growth and glycolysis by targeting miR-4290 and PDK1	Qian et al. (2021)
BDNF-AS	lncRNA	up	WDR5/FBXW7/VDAC3	Promotes progression and inhibits ferroptosis by regulating the WDR5-FBXW7-VDAC3 axis	Huang G. et al. (2022)
circ_0005230	circRNA	down	RHOT1	Serves as a miR-1299“sponge” to enhance RHOT1 expression; promotes GC cell invasion and migration	Peng et al. (2022)

### 4.1 miRNA control of GC through mitochondrial pathways

miRNA are the most studied ncRNAs and play oncogenic and tumor-suppressive roles in GC progression. miR-645 is upregulated in GC tissues and cell lines. Interferon-induced protein with tetratricopeptide repeats 2 (IFIT2) was shown to be a mitochondrial apoptosis protein predicted and verified as the target of miR-645 (Feng et al., 2014). Knockdown of miR-645 increases drug sensitivity and promotes the apoptosis of cancer cells by regulating IFIT2 expression (Feng et al., 2014). The overexpression of miR-18 promotes apoptosis and inhibits proliferation and invasion by targeting hypoxia-inducible factor-1α (HIF-1α) (Wu et al., 2015). The overexpression of miR-18 in tumor cells also activates the expression of mitochondria-mediated apoptosis genes, such as Bax, caspase 3 and caspase 9 (Wu et al., 2015). Overexpression of miR-448 can promote cell proliferation and increase the glycolytic response by inhibiting lysine (K)-specific demethylase 2B (KDM2B) expression. Moreover, Myc proved to be a target of KDM28, which controls the metabolism switch, and miR-488/KDM2B/Myc was a key axis controlling the occurrence and development of GC (Hong et al., 2016). Li et al. (2017) found that miR-148a-3p enhanced the resistance of GC cells to cisplatin by promoting mitochondrial fission and reducing the expression levels of A-Kinase anchoring protein 1 (AKAP1) and DRP1 dephosphorylation. miR-30 was significantly overexpressed in GC tissues and cell lines, and miR-30 inhibition decreased mitochondrial oxygen consumption and activated mitochondria-mediated apoptosis (Wang et al., 2017).

He et al., 2018 found that miR-422a was significantly downregulated in GC, and overexpression of miR-422a inhibited the expression of pyruvate dehydrogenase kinase 2 and affected the cell cycle, ultimately inhibiting cancer cell proliferation. Overexpression of miR-494 sensitized GC cells to TNF-related apoptosis-inducing ligand (TRAIL)-induced cytotoxicity. Overexpression of miR-494 promoted TRAIL-induced mitochondrial collapse and apoptosis pathways by targeting survivin (Xu et al., 2018). Yan et al., 2018 found that miR-4295 could target leucine-rich repeats and immunoglobulin-like domain 1 to activate the EGFR/PI3K/Akt signaling pathway and promote GC cell proliferation. Lee et al., 2019 found that miR-193a-3p could regulate the resistance of GC through the mitochondrial apoptosis pathway, and the peroxisome proliferator-activated receptor γ coactivator 1α (PGC-1α) mediated mitochondrial biogenesis. miR-128 could affect the expression of PGC-1α, and the knockdown of PGC-1α inhibited cancer cell metabolic activity and increased apoptosis (Wang et al., 2019). miR-6778-5p could regulate its host gene SHMT1 by targeting YWHAE to affect the mitochondrial carbon metabolic pathway (Zhao M. et al., 2020). miR-532-3p can inhibit GC cell proliferation *in vitro* and *in vitro* by activating the ARC/Bax/mitochondria-mediated apoptosis pathway (Chen Z. et al., 2021).

### 4.2 Other ncRNAs regulate GC through mitochondrial pathways

Studies have shown that lncRNAs are aberrantly expressed in GC tissues, suggesting that lncRNAs could serve as the target candidates for tumor diagnosis and therapy. LncRNA DLX6-AS1 is overexpressed in GC and DLX6-AS1 knockdown inhibits tumor growth and aerobic glycolysis by targeting miR-4290 and 3-phosphoinositide-dependent protein kinase 1 (PDK1) (Qian et al., 2021). Huang G. et al. (2022) found that lncRNA BDNF-AS aggregates WDR5 to mediate the expression of FBXW7, resulting in changes in the transcription of FBXW7, which in turn regulates the expression level of voltage-dependent anion channel 3 in the mitochondrial outer membrane through ubiquitination. The abnormal expression of circRNAs was found to be related to tumorigenesis and the prognosis of GC. Peng et al., 2022 found that circ_0005230 was upregulated in GC tissues, and circ_0005230 knockdown could inhibit GC cell proliferation and mitophagy by regulating Ras homolog family member T1 (RHOT1) expression *via* sponging miR-1299.

## 5 Conclusion

GC remains a serious public health problem associated with high mortality and poor prognosis. Gastroscopy is the usual method of screening for and diagnosing of GC, but it is unsuitable for all cases (Xie et al., 2020). Traditional screening biomarkers for diagnosing GC are often inadequate due to a lack of specificity. It is urgently necessary to find a diagnostic method for GC. Accumulating evidence has demonstrated that ncRNAs participate in tumorigenesis by regulating invasion, proliferation, and migration (Fonseca Cabral et al., 2020; Tan et al., 2020; Lu et al., 2021; Yang et al., 2022). As mentioned previously, ncRNAs are abnormally expressed in GC and their expression is significantly related to patients’ prognoses. Therefore, miRNAs, lncRNAs, circRNAs, and piRNAs may be biomarkers for the diagnosis and/or prognosis of GC. Compared with protein coding biomarkers, ncRNAs have certain advantages as biomarkers for the diagnosis and/or prognosis of GC. First, compared with protein coding mRNAs, ncRNAs are tissue specific. Second, ncRNAs are more stable in various clinical specimens (such as serum, plasma, urine, and gastric juice), which may allow non-invasive GC detection.

ncRNAs can be used not only as potential biological diagnostic indicators but also as therapeutic targets or combined with other therapies to improve their anti-tumor effects. Some studies have shown that RNA-based therapies have good clinical potential because RNA can directly target specific genes and their products (Lin et al., 2020; Wu et al., 2022). At present, some RNA-based therapies approved worldwide target specific genes in liver or muscle cells and change gene expression by injecting siRNA or oligonucleotide chains (Shaw et al., 2022). Since the important role of ncRNAs in GC progression has been fully demonstrated, they are becoming a new class of targets for RNA-based drug discovery. As a therapeutic intervention, ncRNAs have some advantages over mRNAs because they are more diverse. The pleiotropic nature of ncRNAs makes them particularly attractive drug targets for multifactorial diseases. However, there is no ncRNA-based drug for GC. In addition, ncRNA-based therapy poses many problems, including the stability and toxicity of drugs and the need to avoid immune reactions caused by foreign nucleic acids. The means of administration and the efficiency of drugs after entering the body should also be thoroughly considered.

Mitochondrial dynamics are closely related to the activity of neuronal cells, but some studies have shown that phenotypic changes in cancer cells are also affected by mitochondrial dynamic defects (Sehrawat et al., 2016; Shen and Zhan, 2022). The substances required for tumor metabolism include amino acids and are closely related to mitochondrial metabolism. As mediators of energy metabolism and the signaling pathway, dynamic changes in mitochondria can regulate the signaling pathway. Moreover, changes in the mitochondrial respiratory chain can affect electron transport, energy supply, redox state, metabolism, and apoptosis (Xia et al., 2022). At present, there have been studies targeting mitochondria for treatment of cancer. For example, Lin et al., 2013 found that SOCS6 could promote cell apoptosis and increase the accumulation of Bax in mitochondria; it could also control mitochondrial fission by targeting DRP1. Chuang et al., 2020 reported that imiquimod could significantly increase the apoptosis of skin cancer cells by regulating mitochondrial dynamic. However, the FDA has approved few drugs for mitochondrial therapy. The specific molecular mechanisms of mitochondrial therapy still need to be identified. As an independent organelle, the role of mitochondrial dynamics in cancer development is complex; hence, modes of administration that target mitochondria remain to be studied.

In this review, we focused on the functions of ncRNAs in tumorigenesis and tumor suppression *via* the regulation of mitochondria. Although many ncRNAs have been identified, this is only the beginning. The sensitivity and specificity of ncRNA as diagnostic indexes need to be improved. As a therapeutic target, the drug metabolism, pharmacodynamics, biological distribution, and cellular uptake mechanisms of ncRNAs require further research. This study revealed crosstalk between ncRNAs and mitochondria in the progression of GC. Understanding these networks will provide a theoretical basis for discovering new targets for diagnosing and treating GC and will provide a new approach to clinical GC research.
